# Experimental Structural Template on Tensegrity and Textile Architecture Integrating Physical and Digital Approaches

**DOI:** 10.3390/ma18081721

**Published:** 2025-04-09

**Authors:** Zhiyuan Zhang, Salvatore Viscuso, Alessandra Zanelli, Jinghan Chen

**Affiliations:** TextilesHUB Research Laboratory, Department of Architecture, Building Environment and Construction Engineering (DABC), Politecnico di Milan, 20133 Milano, Italy; alessandra.zanelli@polimi.it (A.Z.); jinghan.chen@mail.polimi.it (J.C.)

**Keywords:** textile architecture, structural template, knitting, tensegrity, ultra-lightweight structures, form-finding, Heinz Isler, Frei Otto, physical model, structural optimization, parametric design, recycled material, polyethylene terephthalate

## Abstract

The construction industry is a major contributor to global carbon emissions, driving the need for sustainable solutions. Ultra-lightweight structures have emerged as an effective approach to reducing material usage and energy consumption. This study explores the potential of ultra-lightweight architectural systems through a learning-by-doing methodology, integrating innovative composite materials, PolRe, and knitting techniques to enhance tensegrity structures for sustainable, deployable, and efficient structural designs. Combining physical modeling, inspired by Frei Otto and Heinz Isler, with digital simulations using Kangaroo 2 and Python, this research employs form-finding and finite element analysis to validate structural performance. A 1:5 scale prototype was constructed using a manual knitting machine adapted from traditional knitting techniques. The integration of elastic meshes and rigid joints produced modular tensegrity systems that balance tension and compression, creating reversible, deployable, and material-efficient solutions. This study bridges conceptual aesthetics with structural efficiency, providing a template for sustainable, ultra-lightweight, textile-based structures.

## 1. Introduction

The construction industry accounts for a significant portion of global emissions [1]. To reduce energy consumption, ultra-lightweight structures have been proposed as a solution to minimize structural weight and lower energy demands during construction. In urban environments requiring flexible spaces, deployable ultra-lightweight structures provide sustainable solutions for temporary architecture [2], effectively reducing energy consumption. These structures are ideal for temporary pavilions, event spaces, and cultural facilities, offering flexible designs while minimizing environmental impact and enhancing efficiency.

### 1.1. Efficiency of Structure

Efficient material utilization is key to building ultra-lightweight structures. Heinz Isler explored minimal surface forms through hanging chain models and developed fully compressive shell structures validated through physical modeling [3]. As a pioneer in using physical models for ultra-lightweight structures, he demonstrated their importance in structural feasibility analysis.

Frei Otto investigated natural force paths and minimal surface geometries through soap film models and hanging chain models, proposing a form-finding method based on physical models [4]. His research, inspired by natural biological structures, introduced biomimetic-inspired structural prototypes that broke away from the traditionally heavy architectural forms in history.

The Munich Olympic Stadium project (1972) is a classic example of ultra-lightweight structures. Frei Otto generated conceptual shapes using soap film models, while Heinz Isler and his team further validated structural performance [5]. Before the development of computer-aided design, physical modeling played a central role in verifying architectural forms. These studies laid the theoretical and practical foundation for lightweight construction, advancing hybrid structures that combine tension and compression systems, depending on the physical model studies for both concept generation and validation.

Fuller and Kenneth Snelson, pioneers of tensegrity structures, introduced the morphology of structure, inspiring engineers to reduce weight in ultra-lightweight designs [6,7,8,9]. By combining continuous tension and discontinuous compression, they created highly stable structural units. Tensegrity structures are valued for their energy efficiency, deployability, and simplicity in aesthetics, making them ideal for deployable, ultra-lightweight structures.

The key defect of traditional tensegrity structures, that they are prone to progressive collapse [8] when the cables fail locally, has not yet been completely solved. When the tension of a cable exceeds its bearing capacity, the entire structure often collapses. In order to enhance the stability of the system, existing studies generally adopt a modular design, add more cables, or monitoring systems to disperse and monitor stress in real time [9]. However, only a few studies have explored replacing conventional single-cable tensile members [10,11,12]. In this context, our work proposed the use of knitted tensile members as a novel alternative. By employing interlock double knitting [13], these elements generate internal friction among the threads [14,15], which helps prevent localized failure and improves overall structural stability.

In recent decades, tensile structures have predominantly employed membrane components; however, the coatings applied to these membranes can impose significant environmental burdens [16,17]. As a sustainable alternative, the integration of mesh components as tensile members in ultra-lightweight architectures has been proposed. Typically, these mesh materials are derived from recycled plastics, such as polyethylene terephthalate (PET) [18], or from bio-based sources, such as fungal networks [19]. Knitted mesh is a mesh material produced using knitting techniques, wherein the interweaving and entanglement of yarns result in a structure that is both elastic and flexible. Unlike traditional woven fabrics or membrane materials, knitted mesh exhibits high malleability and self-adaptive performance, allowing its structural properties to be tailored by varying the knitting density and stitch pattern which offers the distinct advantage of a customizable grading system tailored to specific structural requirements. In this context, the Centre for Information Technology and Architecture (CITA) has implemented Computer numerical control (CNC) knitting technologies [20,21,22] to optimize textile performance, reduce material waste, and fully exploit the material’s intrinsic properties, thereby advancing the potential of textile-based structural systems.

In our research, we employed recycled and recyclable material based on polyethylene terephthalate (PET) inner core and fabric coating supplied by PolRe^®^ Co—a material that offers significant ecological advantages over conventional lightweight alternatives [23]. Recent developments in lattice architecture [24,25] have shown that low-cost materials, when processed via additive manufacturing with precise local control of cell geometry, can exhibit enhanced mechanical properties. In such mechanical metamaterials, the macroscopic behavior differs substantially from the properties of the base material. Our work leverages the inherent flexibility and strength of PET by employing knitting methods to form a loose mesh. This structure is capable of three-dimensional deformation, enabling adaptive changes that create a continuous elastic surface—an important member of the knitted tensegrity system.

### 1.2. Case Study

#### 1.2.1. Frei Otto and His Model Studies

Frei Otto’s contributions to architectural design are rooted in conceptual form generation through physical modeling. He is widely recognized for pioneering soap film models and hanging chain models, which demonstrate minimal surface geometries and force flow patterns:

- Soap Film Models: Otto used soap films to explore tensile equilibrium and minimal surfaces, creating forms optimized for material efficiency and structural performance.
- Hanging Chain Models: Inspired by Gaudí [26], Otto employed hanging chains to study compression-free forms, later inverting these models to develop self-supporting geometries suitable for large-span structures.

These experimental methods allowed Otto to generate organic forms that closely followed natural force flows, aligning with his philosophy of “form follows force”—an approach that continues to influence modern parametric design.

#### 1.2.2. Heinz Isler and Physical Model Validation

In contrast to Otto’s focus on conceptual exploration, Heinz Isler emphasized the validation of structural performance through physical testing:

- Shell Structures: Isler’s primary focus was on compression-only forms, which he developed using hanging membrane models. These models, when inverted, produced structurally efficient shells optimized for minimal material use.
- Buildability Testing: Isler’s method demonstrated that physical models could serve not only as form-finding tools but also as engineering validation frameworks, ensuring the constructability of the proposed forms.

Isler’s process bridged the gap between conceptual design and practical implementation, proving that thin-shell structures could achieve strength and efficiency through proper material and form distribution.

#### 1.2.3. Munich Olympic Stadium: Integration of Tension and Compression

The Munich Olympic Stadium represents a landmark project where tension and compression systems were combined to create a hybrid structure.

Initiated by Frei Otto, he used foam film models to generate the shape, capturing the fluid, organic form of tensile membranes. He then developed hanging chain models [4,5] to study the equilibrium of forces, ensuring that the geometry followed natural force paths.

At a time when computer-aided design tools were not yet available, Isler and the engineering team validated the structure’s stability and feasibility through physical modeling [3] and stress testing.

The result was a lightweight, transparent roof structure that embodied both conceptual innovation and engineering rigor. It became a benchmark for integrating design and performance through physical models.

#### 1.2.4. Key Insights from Physical Models

Key insights included the following:

1. Natural Force Paths: Physical models allow designers to visualize force distribution intuitively, revealing tension and compression zones in a tangible way;
2. Material Efficiency: These models inherently encourage minimal material use, ensuring structural optimization without excess weight or waste;
3. Practical Feasibility: Before the advent of advanced computational tools, physical prototypes provided the primary method for validating form-finding processes and structural performance.

## 2. Methodology

### 2.1. Research Objectives

This study ultimately aimed to develop an ultra-lightweight, deployable, and efficient structural template through a learning-by-doing approach. This template not only characterizes the geometric configuration but also provides both theoretical and practical foundations for subsequent design optimization and full-scale engineering applications. By combining physical modeling and digital simulation tools, this research explores the structural performance of knitted PolRe^@^ materials provided by PolRe Textile Innovation S.r.l in Mantova, Italy.

The design process is further refined through a scale of 1:5 and physical model research, deepening the exploration of details and structural performance.

The digital simulation process adopts finite element analysis using digital tools Grasshopper 1.0.0008, Python 3.13, and Kangaroo 2.

**Figure 1:**
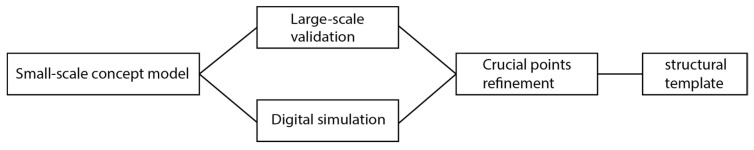
The working flowchart.

The research process is structured as it is shown in Figure 1:

1. Exploring Knitting Techniques

Through experimentation with various techniques on ropes and threads, the benefits of using knitting to create elastic mesh surfaces became apparent. Knitted mesh shows great potential as an ultra-lightweight structural component due to its adaptability, strength, and inherent elasticity.

2. Concept Model Development

A small-scale conceptual model was developed using a nylon elastic mesh. This initial phase focused on understanding the material’s behavior and its potential integration into architectural functions.

3. Validation of Buildability at 1:5 Scale

To validate the structural buildability of the template, a larger model was constructed using PolRe, the material under study. This prototype, built at a 1:5 scale, was produced with a scaled-up, manual knitting machine inspired by traditional knitting looms. This process demonstrated the feasibility of creating elastic mesh structures at an architectural scale.

4. Digital Analysis for Structural Performance

To better understand the structural performance of the templates, finite element analysis was employed. Digital tools, including Grasshopper 1.0.008, Python 3.13, and Kangaroo 2, were used to analyze the templates, focusing on tension distribution, deformation, and overall stability under various loads.

5. Critical Evaluation and Refinement

By combining insights from physical models and digital simulations, the study identified critical areas within the structure. These weak points were analyzed in detail to propose enhancements in both material use and structural design, ensuring better buildability and performance.

This iterative approach allows the research to bridge the gap between conceptual exploration, physical validation, and computational optimization, ultimately leading to the development of scalable, efficient, and sustainable, ultra-lightweight structural templates.

### 2.2. PolRe as a Material

The inherent characteristics of PolRe guide and influence the design process, particularly its stiffness, lightness, and elasticity achieved through knitting. These properties are harnessed to create ultra-lightweight and deployable structures.

The PolRe material comprises two main parts:

- An internal support structure made of a moderately flexible polyethylene terephthalate (PET) [27];
- An external fabric coating, whose primary function is to protect the PET core from heat and UV-induced aging.

As it is shown in Table 1, unlike the other conventional materials for textile architecture, such as PVC and PTFE, which are generated from non-recycled resources, PET is highly recyclable. It minimizes environmental footprints and maintains a high tensile strength and durability.

When the structure is placed under axial load, the external coating does not bear any load. Hence, for the purposes of this study, we focus on the physical properties of the PET core, which carries the primary structural load.

In our application, the PET materials employ two widths, 4 mm, and 8 mm, both with a thickness of 1 mm, resulting in a semi-rigid strap that can bend around edges yet still provide substantial tension capacity. The key physical properties of these PET straps include the following:

Owing to PET’s favorable mechanical attributes—including creep resistance, fatigue resistance, low friction, and stability over a broad temperature range—PET-based hybrid materials present notable potential for tensile architectural applications. However, the core exhibits brittleness and a tendency for stress concentration, particularly at joints. To mitigate this, minimizing the use of joints in designs enhances structural integrity.

PolRe’s variable knitted outer layer allows for customizations in color, texture, and sheen to meet specific design requirements. Due to its flexibility and resilience, PolRe’s design exploration begins with forming planes from lines. The arrangement of lines within the plane enables the creation of unique textures and translucencies, adding visual depth to structures. PolRe offers three primary methods for mesh construction: weaving, winding, and knitting. Among these, knitting emerges as the most advantageous due to its flexibility and ability to maintain structural strength while reducing stress concentrations by reducing joints.

### 2.3. Knitting as the Optimal Approach

Knitting provides a flexible surface that is stretchable in multiple dimensions and requires only two nodes at the endpoints, reducing potential stress points. By varying stitching techniques, the mesh’s performance can be customized [13,20]. Traditional knotless netting methods inspire innovations in bespoke meshes, enhancing effectiveness and adaptability. By investigating PolRe’s physical and mechanical properties, emphasizing its sturdiness and resistance as pivotal factors for applications in ultra-lightweight architecture, studying physical models of PolRe enhances the understanding of its unique characteristics and informs its design potential. The exploration of knitting techniques demonstrates the material’s flexibility and its ability to create efficient and adaptable meshes for architectural use. This research aimed to develop a validated structural template based on knitted PolRe materials, emphasizing a deployable and reversible design with scalability potential.

Since the physical performance of knitting PolRe mesh has a big unknown, a research study based on the physical model study has been conducted. The following sections describe a practical case primarily based on physical model studies, aimed at validating the buildability of ultra-lightweight, innovative structural applications.

## 3. Results

### 3.1. Physical Models

**Figure 2:**
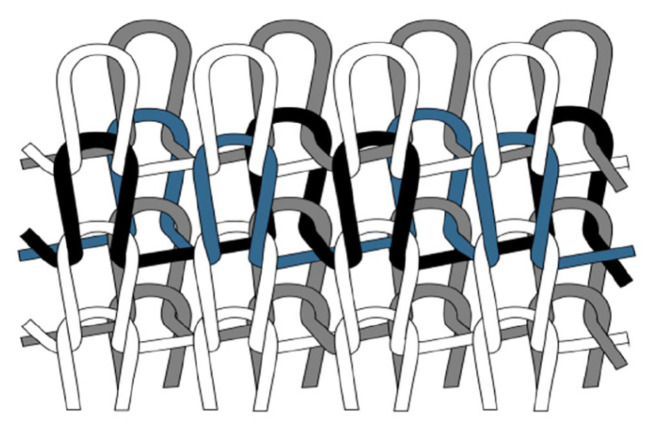
Interlock double knit pattern (*Journal of Engineered Fibers and Fabrics*).

Knitting represents a promising fabrication technique for creating tensile meshes, offering potential not only as a tensile element within knitted tensegrity systems but also as a strategy to mitigate progressive collapse through innovative methods such as interlock double knitting [13] (Figure 2). To streamline the scope of this study, detailed discussion of specific knitting patterns is intentionally omitted.

Given the inherent complexity of the geometry and the nonlinear structural behavior under tension, existing computational tools remain insufficient to fully capture the performance of these structures or yield robust quantitative results. Consequently, physical modeling is adopted as the primary method for validating structural feasibility. These models serve multiple critical functions: they generate preliminary structural templates, a knitted tensegrity unit; inform functional architectural applications; and facilitate detailed node-level design. This iterative process of physical testing and refinement not only enhances our understanding of the structure’s real-world behavior but also provides essential feedback for improving computational analyses and predictive models.

#### 3.1.1. Conceptual Physical Models

**Figure 3:**
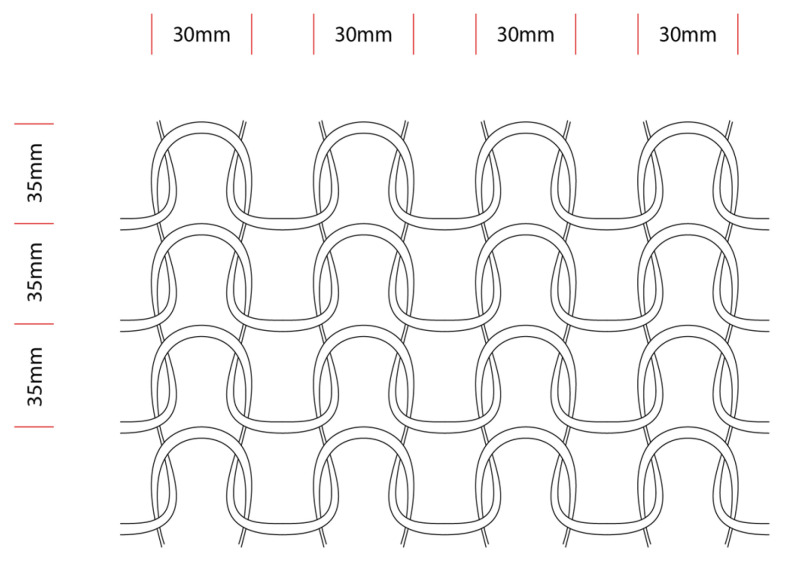
The dimensions of the loops.

In our research, we adapted the simple knotless netting pattern, half-hitch (Figure 3) around half-hitch for testing and form-finding.

1. Knitted Mesh for Tensile member

**Figure 4:**
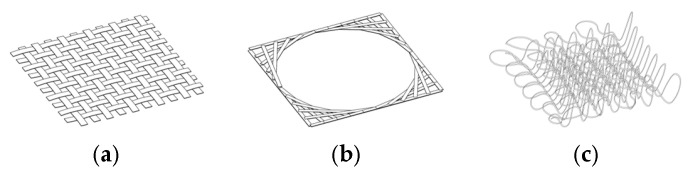
Different mesh forming methods with PolRe: (**a**) flat weaving mesh; (**b**) flat winding mesh; (**c**) 3-dimensional knitted mesh.

Compared to other techniques that form mesh structures from linear components (Figure 4), knitting with PolRe threads produces a three-dimensional loose mesh. This distinct configuration arises from the inherent flexibility and resilience of the PolRe threads, resulting in emergent properties, particularly in form–formation behavior, that differ from those of the raw PolRe material [24].

**Figure 5:**
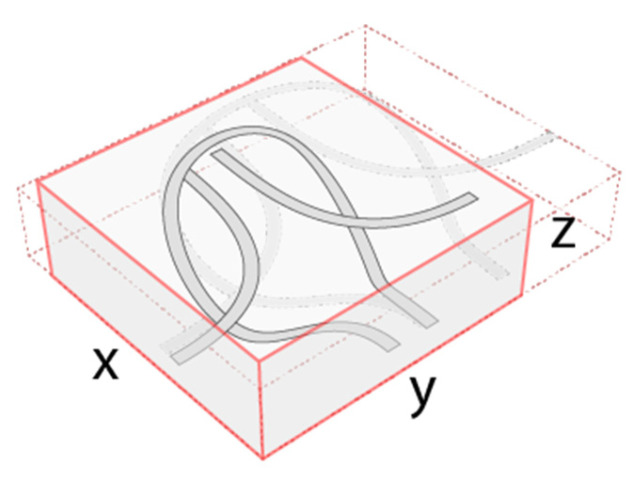
A diagram of a 3-dimensional stitch with PolRe.

As it is shown in Figure 5, the dimension of a stitch depends on x, y, and z, three values; by stretching the mesh, the z value decreases, and x and y increase, resulting in the elongation of the knitted mesh.

**Figure 6:**
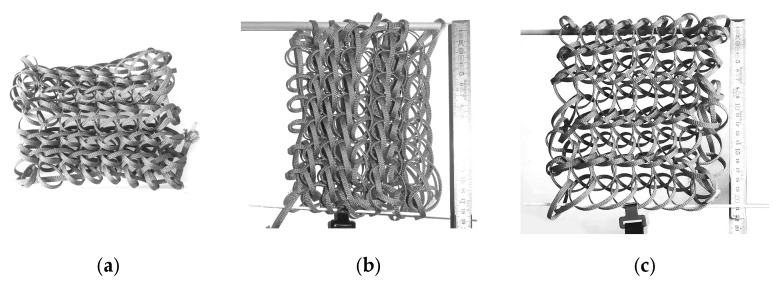
The deformation features of the mesh: (**a**) a knitted PolRe mesh in a loose state; (**b**) stretch the mesh in a horizontal direction; (**c**) stretch the mesh in a vertical direction (the row spreads almost parallel).

To elucidate the mechanism governing the mesh’s behavior, we conducted a non-destructive test to predict its elongation. Sample meshes (Figure 6) were fabricated using an 8 × 9 stitch configuration of PolRe threads (4 mm wide and 1 mm thick), resulting in an overall mesh approximately 190 mm in width, 165 mm in height, and 30 mm in thickness.

F = a⋅(ΔL)^b^

**Figure 7:**
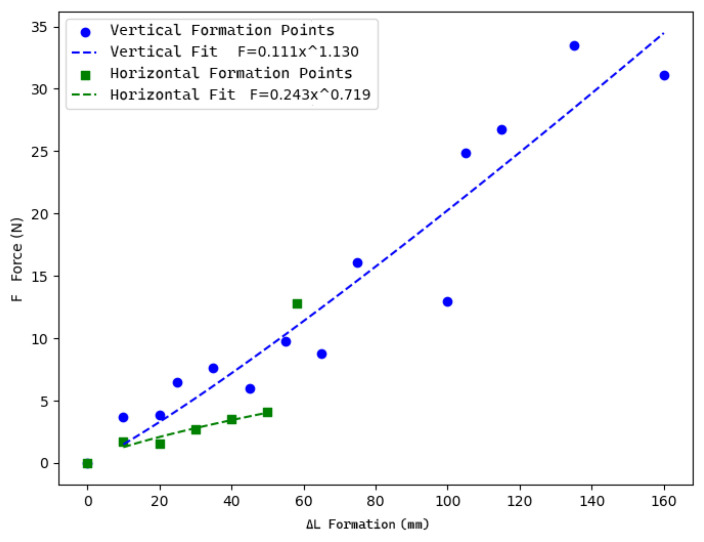
Nonlinear fit for mesh tensile behavior.

Based on multiple measurements of mesh deformation elongation, we applied a nonlinear fitting method using the model (Figure 7) where ΔL represents the formation and F is the corresponding force. In the horizontal direction, due to constraints imposed by the weaving nodes, the formation remains below 30% and follows a linear trend—excluding the final data point that reached the non-destructive test limit. In contrast, the longitudinal direction exhibits a formation of nearly 97%, displaying a nonlinear response.

Based on the experimental results, it can be inferred that the nonlinear behavior observed in the longitudinal direction is likely due to the friction [14,15] generated by the PolRe fabric coating during knitting, coupled with the inherent sturdiness of the inner core PET material. However, the mechanical properties of the mesh remain closely tied to the mesh density and the specific knitting method employed; as the number of stitches increases, a greater force is required to achieve the same level of deformation. Given the challenges in accurately predicting the nonlinear behavior of tension structures using current computational tools, these experimental data serve as a critical empirical foundation for developing physical models and calibrating numerical simulations. Furthermore, by comparing the deformation characteristics across different orientations, the reliability of the performance prediction methods based on the physical experiments can be enhanced and validated in future studies, thereby providing valuable data support for subsequent in-depth analyses.

2. Structural unit and Form generation

**Figure 8:**
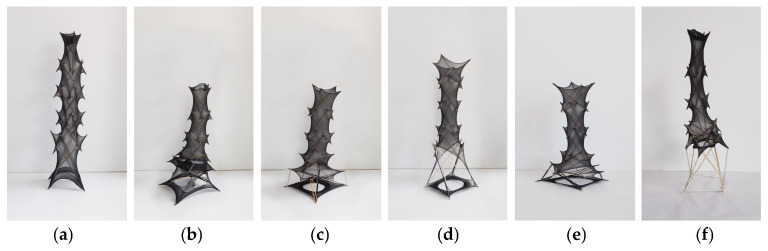
The physical iterating of the openings in the conceptual model. (**a**) A continuous mesh integrated with isolated compression rods; (**b**) Replace the bottom unit with a separate open mesh; (**c**) A continuous mesh with the bottom unit open, additional tensile added to provide the tension; (**d**) A continuous mesh with the bottom unit open; (**e**) A continuous mesh with the bottom unit open, additional tensile added to provide extra tension; (**f**) A conventional tensegrity unit integrated with continuous meshed units.

The structural unit is an advancement based on the tensegrity structures created by Kenneth Snelson. In traditional tensegrity structures, a single continuous wire connects the rods, creating a lightweight visual effect. However, the drawbacks for progressive collapse become a barrier for the structure to be applied widely. Below the experiments (Figure 8b,d) show that, by enveloping the rods with a tensioned, interlock nylon mesh instead of wires, even if one strand in the mesh breaks, the friction maintains its stability to a certain extent, thus making the structure more robust and reliable.

The idea of this design is to create a tensioned structure tower enveloped in mesh by stacking the developed units. The base layer is enlarged to accommodate human scale (Figure 8a). The height of the tower can be adjusted by varying the number of units according to different functional needs. This design approach starts from defining the structural form from the bottom up. Below are iterative schemes outlining the integration of architectural applications with the structural template (Figure 8b–f). Table 2 summarizes the spatial comparison results.

3. Architectural Application Prototype development

**Figure 9:**
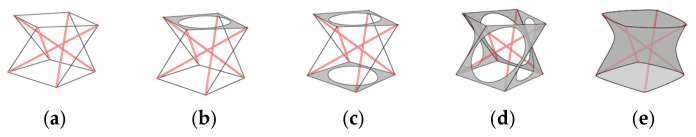
The various iterations of continuous tensile mesh (or cables) combined with discontinuous rods: (**a**) Type a: a conventional tensegrity unit; (**b**) Type b: a unit in which the top surface and its edges are replaced by a tensile mesh featuring a central aperture; (**c**) Type c: a unit where both the top and bottom surfaces, along with their edges, are substituted by tensile meshes, each with a central aperture; (**d**) Type d: a unit in which all surfaces are replaced by meshes with a central aperture; (**e**) Type e: a unit where all discrete cables are replaced by a single, continuous mesh.

After several trials, we found that Model d Figure 9 exhibits the most design coherence that can be translated into a full-scale structure using a bespoke knitted component.

#### 3.1.2. Scaled Model Built up with Manual Knitting Machine

**Figure 10:**
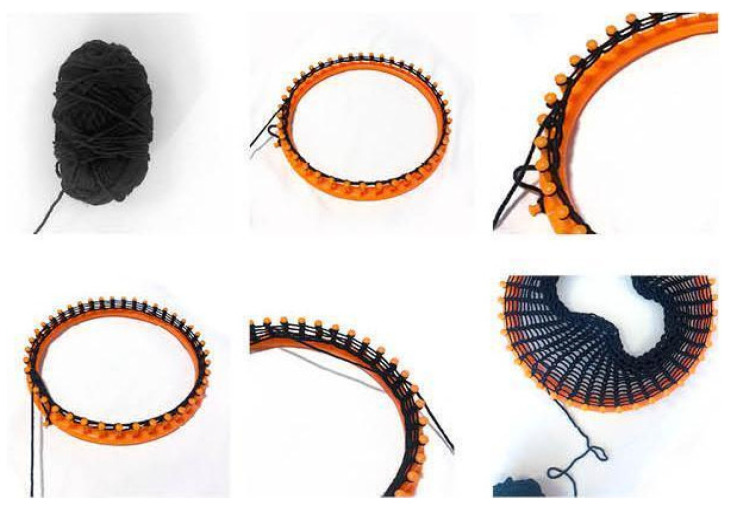
An effective knitting method through spool knitting.

**Figure 11:**
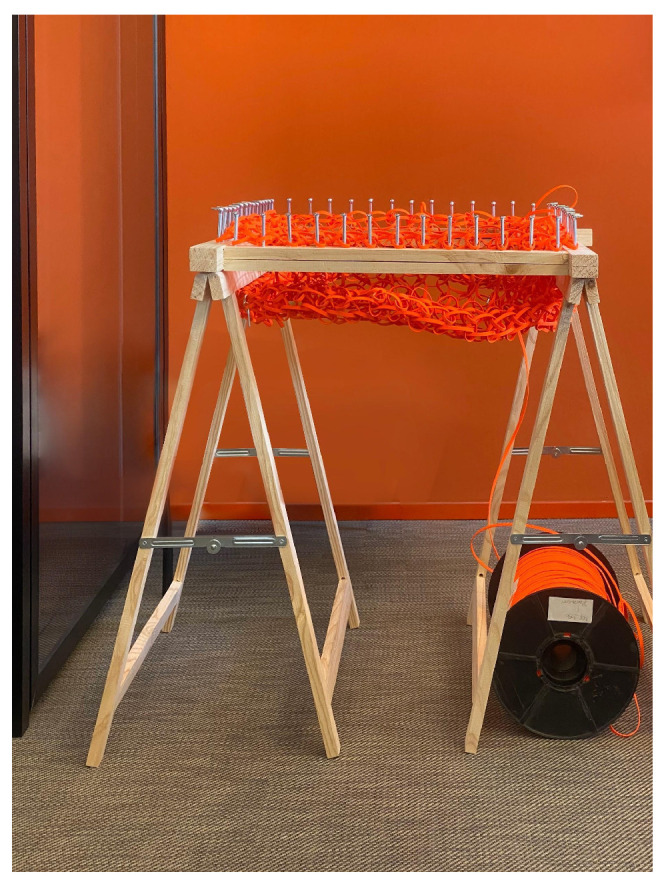
A piece of mesh under knitting on the knitting machine.

To streamline the knitting process with PolRe, we explored traditional knitting techniques and adopted the spool knitting method (Figure 10) [28]. The forming method involves wrapping yarn around the pegs and then lifting the lower loops over the upper loops to form stitches. This technique allows the threads to be configured not only into tubular shapes but also into flat fabrics. As a result, by scaling up this traditional concept, we can create an efficient and evenly distributed PolRe mesh pattern (Figure 11).

**Figure 12:**
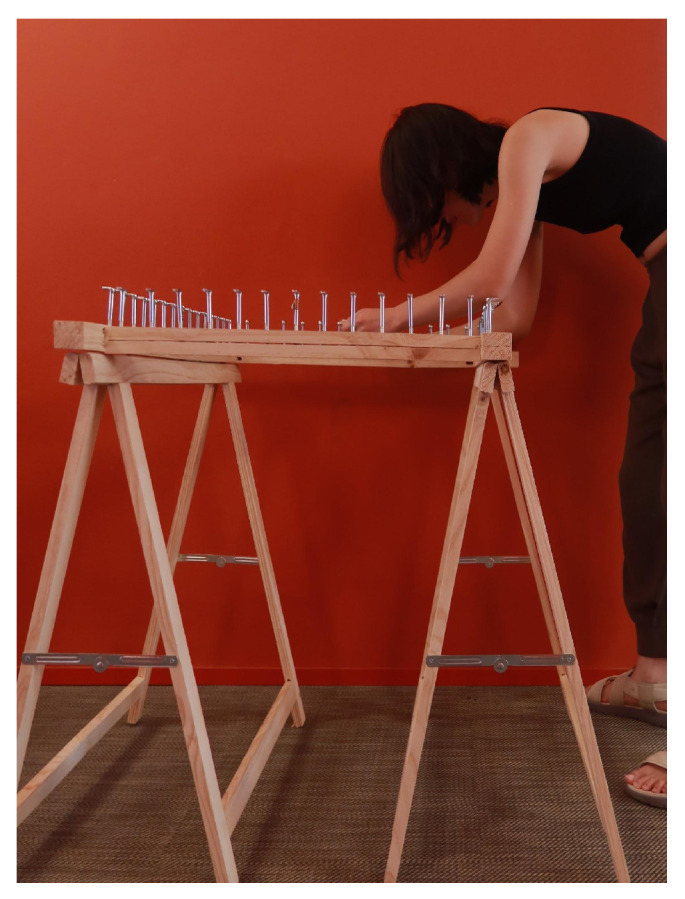
A knitting machine inspired by spool knitting.

The machine (Figure 12) features a square frame measuring 50 cm × 60 cm, which is suitable for producing larger fabric panels. Standing at a height of 75 cm, for comfortable use while seated, the structure is built from sturdy wood to ensure stability and durability, with 56 evenly spaced metal screws around the rim serving as knitting pegs.

#### 3.1.3. Knitting Process

**Figure 13:**
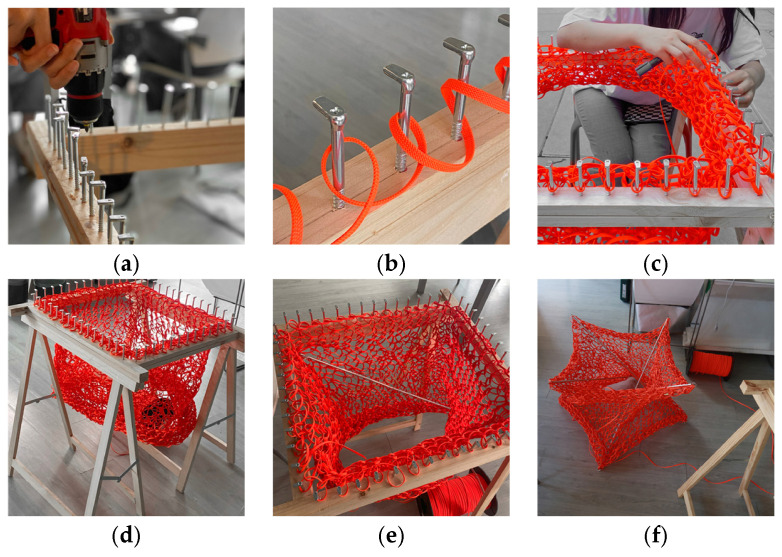
Knitting process with PolRe: (**a**) Building the knitting machine with even screws; (**b**) wrapping yarn around the pegs; (**c**) lifting the lower loops over the upper loops; (**d**) a complete unit of mesh; (**e**) insert aluminum rods to stretch the mesh; (**f**) a complete knitted tensegrity unit.

As it is shown in Figure 13, the custom manual knitting machine, constructed from a sturdy wooden frame with 56 evenly spaced metal screws, forms the foundation for the knitting process. Each screw functions as a peg, securing the yarn as it is looped to create the mesh. As additional rows are added, the mesh begins to curl inward due to the sturdiness of the inner core material and the knitting pattern’s direction.

A complete unit of the knitted mesh unit exhibits a consistent, uniform pattern that satisfies both structural and aesthetic criteria. Hooks are affixed to each corner, enabling the insertion of 1 m-long aluminum rods (8 mm in diameter) at regular intervals—every 28 stitches in the vertical direction and every 14 stitches in the horizontal direction along the boundary (before the rods begin to bend). These rods exert tensile forces that counteract the mesh’s compressive restorative force, thereby establishing a spatial structure within the members.

**Figure 14:**
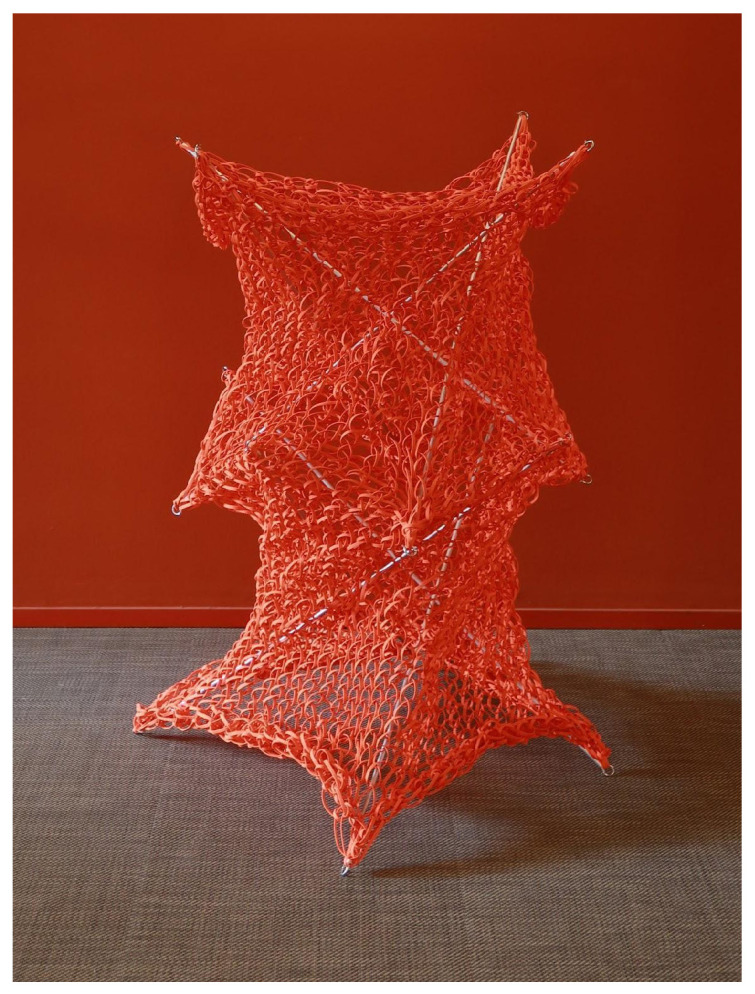
Two units of knitted tensegrity system.

The process is repeated, resulting in a two-unit knitted tensegrity structure, as illustrated in Figure 14.

**Figure 15:**
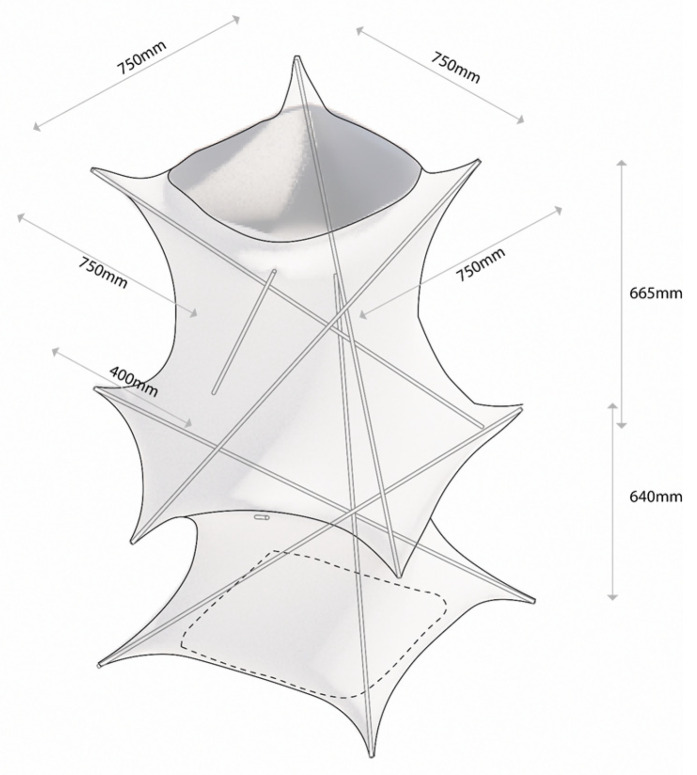
Diagram of model dimensions.

As it is shown in Figure 15, the circumference at the maximum diameter measures approximately 300 cm, while that at the minimum diameter is about 160 cm. The height of the lower monomer is 64 cm, whereas the upper monomer reaches 66.5 cm. This discrepancy stems from the self-adaptive behavior of the structure, wherein the lower monomer experiences a greater self-weight.

In the relaxed state, measurements indicate that the mesh circumference is around 133 cm. Under the boundary conditions of the physical model (before the rods start to bend), the elongation at the minimum diameter is about 20%, whereas at the maximum diameter it is roughly 125%. This latter value exceeds the 96% vertical-direction elongation observed in plane mesh tests, likely because the structure deforms concurrently in both horizontal and vertical directions.

### 3.2. Digital Simulation

**Figure 16:**
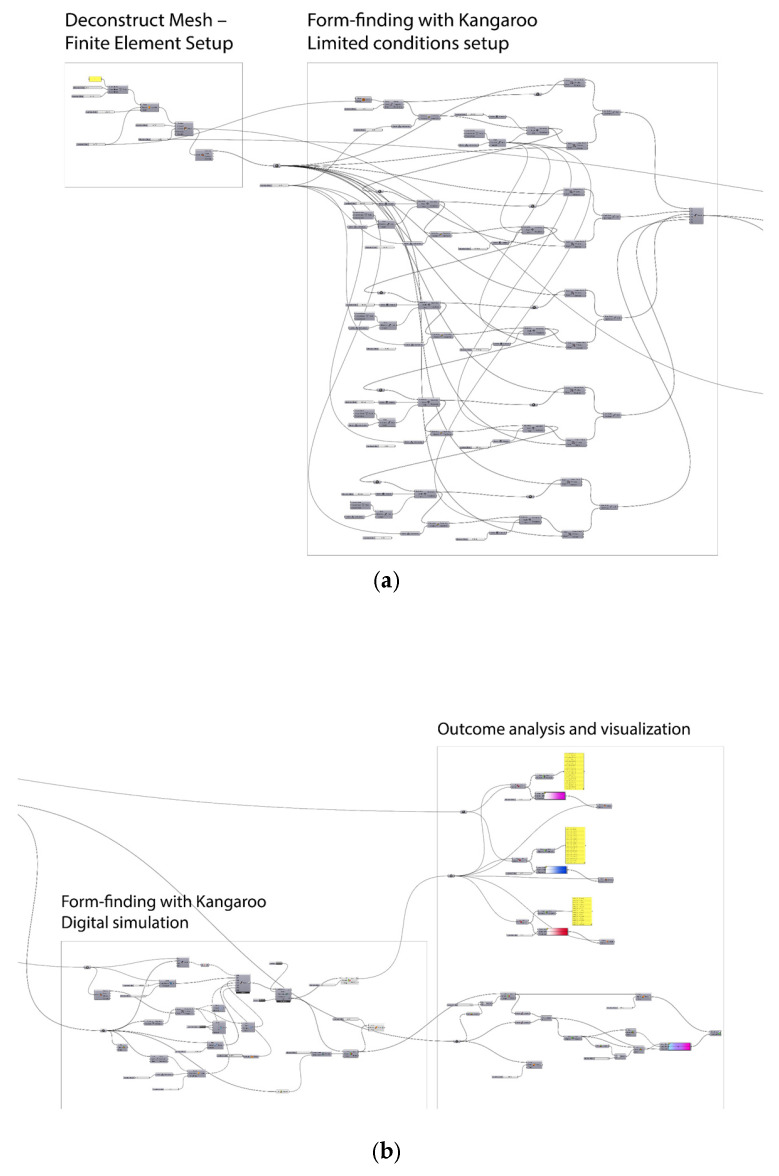
The digital simulation example of the tension forces analysis with Grasshopper 1.0.0008. (**a**) Establishing the boundary conditions using the geometric model and supporting rods; (**b**) conducting form-finding simulations with Kangaroo 2, followed by digital simulations using Python 3.13.

To optimize the knitted mesh and its connections to the supporting rods, a force analysis was conducted using Grasshopper 1.0.0008 (Figure 16) on a three-unit assembly, examining the structural performance at the top, middle, and bottom sections. Due to the inherent variability and uncontrollability observed in the test results for the knitted mesh, we have used the tension data from the supporting rods—under the specific condition that no bending occurs (maintained at a fixed length of 1000 mm)—as our boundary condition. This approach isolates the tensile behavior of the system, providing a consistent and repeatable benchmark for calibrating our digital model. By aligning the numerical simulation parameters with the empirical tension measurements from the rods, we ensure that the digital model reflects the essential mechanical response of the structure. While this boundary condition simplifies the complexity inherent to the knitted mesh, it serves as a practical basis for our initial calibration efforts.

**Figure 17:**
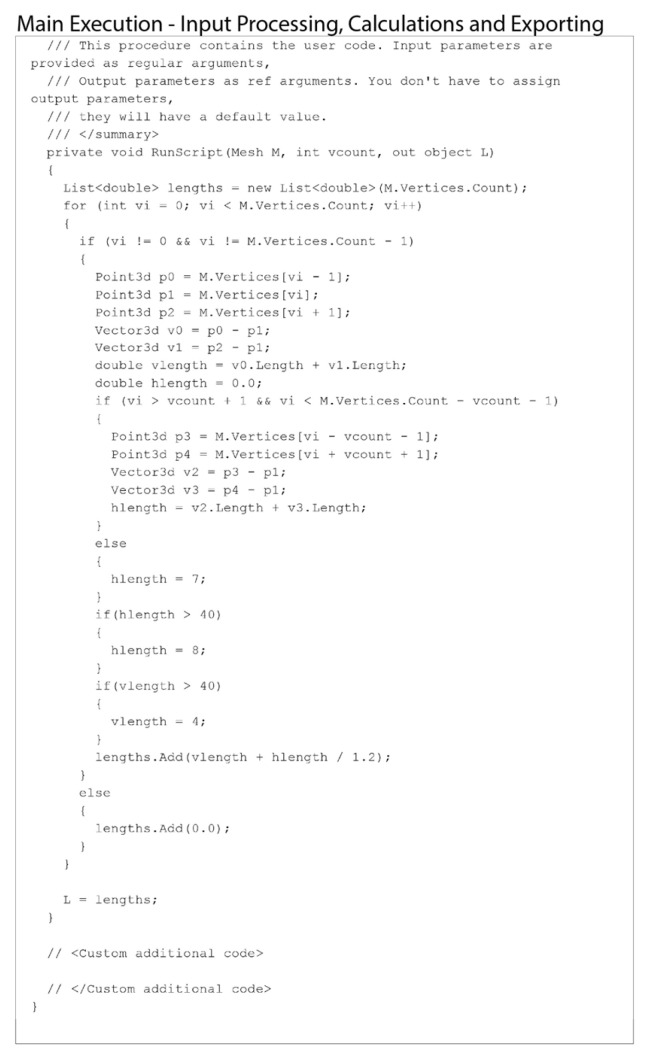
The main Python 3.13 code used for analyzing the tensional force in the mesh by calculating the elongation value.

It is worth noting that there are currently no readily available Grasshopper 1.0.0008 plug-ins to perform nonlinear behavior in the tensile condition. To overcome this limitation, we developed a custom C# code to conduct the simulations (Figure 17) by calculating the elongation value.

The tension is calculated by measuring the distances between each vertex and its neighbors (above/below or left/right). These distances are summed to determine the total vertical tension at each vertex. The results remove the abnormal values from the tips of the mesh, where the tensile mesh meets with the compressive rods.

**Figure 18:**
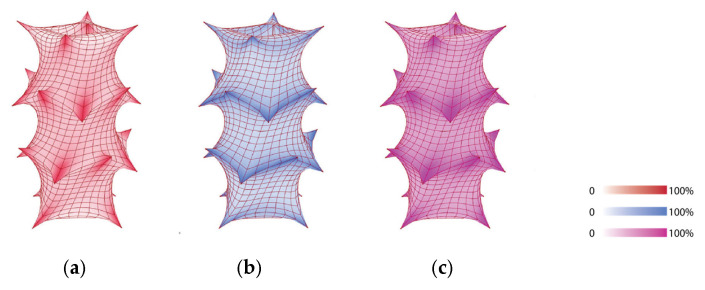
Digital simulation results using Grasshopper 1.0.0008 and Python 3.13: (**a**) tension on the vertical dimension; (**b**) tension on the horizontal dimension; (**c**) tension on both dimensions combined.

In the digital simulation results with Python 3.13 (Figure 18), the overall outcomes align closely with the physical model’s behavior. The mesh undergoes its greatest stretching at the joints and along the lines connecting them. We excluded anomalously high values at the mesh tips, as a perfectly sharp corner does not exist in reality. Another discrepancy is that, in the digital model, the horizontal elongation slightly exceeds the vertical elongation, whereas the physical model shows the opposite. We hypothesize that this occurs because the digital model treats the mesh as a smooth membrane plane, neglecting the friction between threads inherent in the knitting process.

## 4. Discussion

### 4.1. Scaled Physical Model Node Refinements

In this section, this study focuses on refining the structure by addressing its weak points through an evaluation of the physical model at a 1:5 scale using PolRe material, alongside digital simulation results (Figure 18). After constructing the morphology of the structure, the mesh was stretched and fitted onto the rods, deforming it into a new, stable shape. In this new configuration, the joints between the rods and the flexible mesh emerged as the most rigid and deformed areas, highlighting their critical role within the structure. This observation was further validated and visualized through digital simulations, offering a more parametric understanding of the forces at play. To improve the structure, three enhancement methods were proposed: first, tying more stitches with the hooks and, second, reinforcing the knitting pattern around the joints using crochet techniques. A 1:15 model with cotton yarn was made to understand the feasibility and, third, incorporating rigid holds to create more secure and stable connections between the mesh and the rods.

1. Simple Solution on the physical model

**Figure 19:**
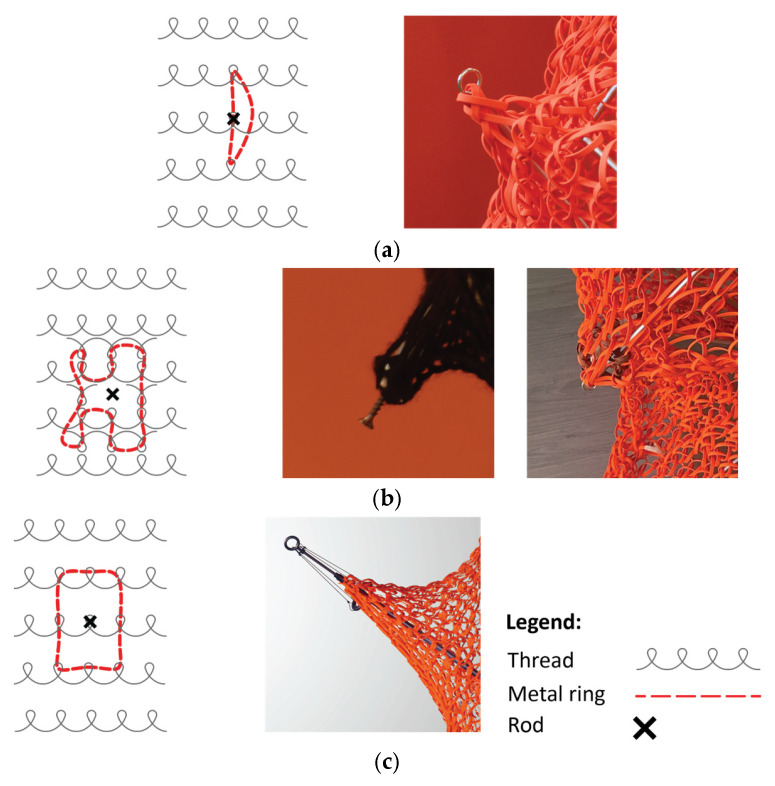
Different node-strengthening method diagrams with models. (**a**) Diagram and physical model of the simple solution on the connected thread; (**b**) diagram and physical models of the crochet-based node; (**c**) diagram and digital model of the final node design.

The first study focused on the natural outcomes from the 1:5 scale morphology model (Figure 19a). By tying three vertical lines of knitting, the nodes demonstrated stability and effectively held the mesh in place.

2. Crochet-Based Node

**Figure 20:**
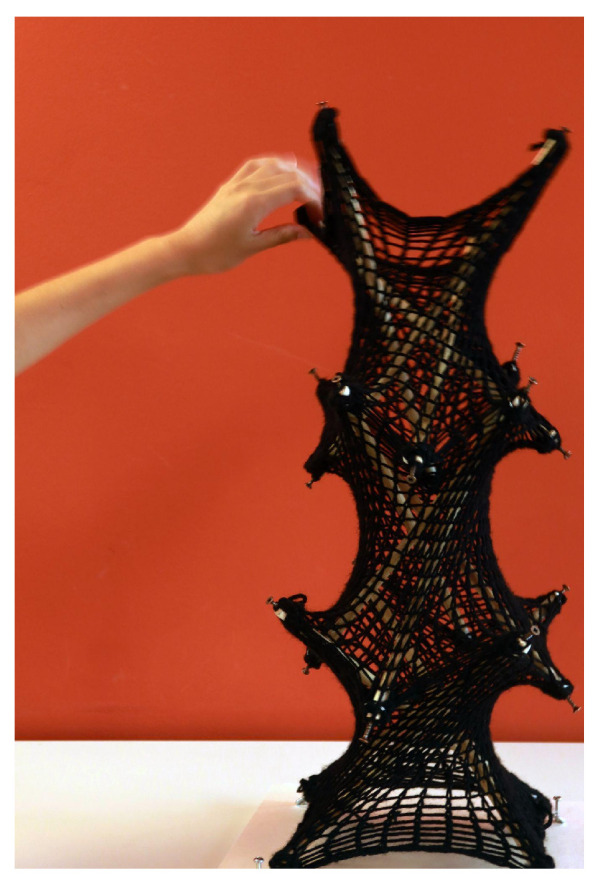
1:15 cotton model with crochet-enhanced nodes.

**Figure 21:**
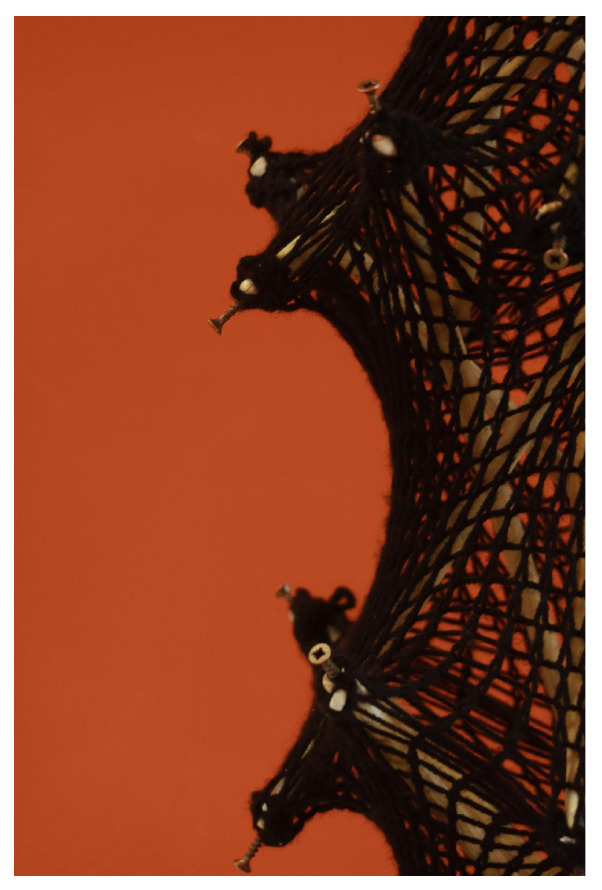
Nodes to thicken the mesh and prevent compressive members from puncturing.

Inspired by the CNC bespoke knitting membrane method brought up by Hybrid Tower by CITA [22], a 1:15 scale model was created (Figure 20) using traditional crochet techniques. This method produced a dense mesh at the connection points (Figure 21), which worked effectively with cotton yarn on a smaller scale. However, when applied to the PolRe material in the larger model, the technique proved redundant and less efficient due to the stiffness of the material.

3. Final Node Design

The final node design emphasizes simplicity and practicality (Figure 19c). Compared to crochet nodes, rigid rings are introduced to connect 3 × 3 rows of eight stitches along the mesh edges, enabling forces to distribute evenly in all directions. This method reduces concentrated pressure at single points, spreading it across a larger surface area to improve stability and durability.

Informed by insights from previous studies, the final node design achieves a balance between structural stability, aesthetic appeal, and ease of manufacturing. This refined solution ensures the nodes perform effectively while aligning with the project’s architectural vision. Moreover, the design facilitates the use of modular, separated rod units, which ensures rapid assembly and disassembly while aligning with the project’s overall architectural vision and practical construction requirements.

### 4.2. Implications and Challenges

The rise of digital technology has significantly advanced and validated the application of conceptual structures in architecture. Historically, architects relied on physical methods to verify the efficiency and feasibility of building designs. Today, digital tools have become the dominant medium for architectural design, offering new opportunities for precision and optimization. However, it is essential to balance digital innovation with an understanding of material properties and structural principles to ensure both design efficiency and rationality alongside aesthetics. In our case of study, the complexity of the mesh behavior, including the composite material, mechanism of knitted PolRe, and nonlinear behavior on the tensile mesh surface, made it impossible to obtain precise quantitative results as the conventional structural design. Therefore, by looking back to history, going back to the physical model, we will be able to move forward in the research path. By integrating digital and physical models, designs and nodes can be refined across different scales.

Despite the advancements, challenges remain. For example, while Grasshopper offers valuable tools for design, it lacks comprehensive force analysis capabilities for complex tensile deformation. The integration of Python to calculate the elongation value has partially addressed this gap, but discrepancies between digital models and their physical counterparts persist. Addressing these inconsistencies requires deeper exploration of mesh surface behavior in different directions, material behavior, enhanced data integration, and the use of sensors and other technologies to bridge the gap between digital and physical performance.

## 5. Conclusions

**Figure 22:**
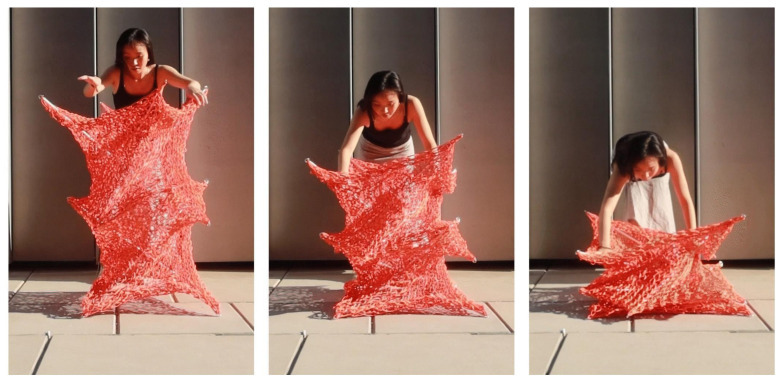
Final outcome of the knitted tensegrity system, which can be folded and carried easily by one person.

This research employs a bottom-up strategy, initiating with a “learning-by-doing” approach using PolRe material and a form-finding methodology inspired by Frei Otto and Heinz Isler. By integrating large-scale physical modeling with digital simulations, we developed and validated a structural template that enhances tensegrity systems through the use of knitted PolRe. The proposed design, characterized by its deployability, reversibility, and ease of construction (Figure 22), represents a novel paradigm in ultra-lightweight architecture. Our findings demonstrate that elastic knitted materials can significantly enhance structural efficiency while minimizing material usage, thereby meeting the growing demand for sustainable construction solutions. Although further research is required to fully understand the complex behavior of knitted meshes, optimize knitting patterns, and enhance full-scale performance, this study provides a robust foundation for future investigations. Refining CNC fabrication techniques and incorporating advanced digital simulations with sensor technologies will be critical to achieving greater precision, efficiency, and scalability, ultimately expanding the architectural possibilities of textile-based systems.

## Figures and Tables

**Table 1:** The mechanical properties of PET.

Chemical Formula	C_10_H_8_O_4_
Density (g/cm^3^)	1.38–1.56
Flammability	Self-extinguishing
Freezing resistance (°C)	−50
Usable max. Temperature (°C)	70
Tensile strength (MPa)	40–60
Young’s modulus (MPa)	1000–3500
Flexural strength (MPa)	55–100
Elongation at break (%)	19–46
Flexural modulus (MPa)	2000–3500
Hardness (Shore-A)	96

**Table 2:** Comparisons with different iterations to generate architectural space.

Model and Type	Description	Advantages	Disadvantages
Figure 8a: Type e	A continuous mesh spans from the bottom to the top, integrated with isolated compression rods.		The entire structure is wrapped in textile, limiting accessibility to the interior space.
Figure 8b: Type d + Type e	Based on Model, but incorporates an isolated tensegrity unit at the bottom with openings on each side.		Although this approach introduces openings, the fabric pieces at the bottom act as tensile members that hinder entry. Moreover, unnecessary separation disrupts the overall aesthetic coherence.
Figure 8c: Type c + Type e	A continuous mesh with openings. The mesh does not provide vertical tension, so additional cables serve as tensile members in place of the mesh in that direction.		The addition of rigid cables results in a redundant design language, and the risk of progressive collapse remains. Furthermore, the full potential of the tensile mesh is not realized.
Figure 8d: Type d + Type e	A continuous mesh with bespoke opening shape that provides tension throughout the entire system	The tensile members carry only tension, while the rods handle compression alone. This clarifies the design language of the structural form, creating a clean tension–compression system.	Because of the uniform knitting pattern, the ground level is looser than the upper levels, rendering it less stable than the original model. Possible solutions involve reinforcing the knitting pattern or adding extra joint elements for better stability.
Figure 8e: Type c + Type d + Type e	Building on Model d, additional cables are added to reinforce the tension within the system.	Offers a strong, stable base at ground level, enhancing the structure’s load-bearing capacity and overall robustness.	Incorporates redundant components that add unnecessary complexity to the design language.
Figure 8f: Type b + Type e	A rigid cable delivers tension for the first unit, and from the second unit onward, a continuous mesh system is used.	Provides a slender, floating appearance, contributing a distinctive aesthetic quality to the structure while maintaining functional integrity.	Does not effectively address the progressive collapse concern.

## Data Availability

The original contributions presented in this study are included in the article. Further inquiries can be directed to the corresponding authors.
